# Fractal lacunarity of trabecular bone in vertebral MRI to predict osteoporotic fracture risk in over-fifties women. The LOTO study

**DOI:** 10.1186/s12891-021-03966-7

**Published:** 2021-01-23

**Authors:** Annamaria Zaia, Roberto Rossi, Roberta Galeazzi, Manuela Sallei, Pierluigi Maponi, Pietro Scendoni

**Affiliations:** 1 Centre of Innovative Models for Ageing Care and Technology, Scientific Direction, IRCCS INRCA, Via S. Margherita 5, I-60121 Ancona, Italy; 2 Medical Imaging Division, Geriatric Hospital, IRCCS INRCA, 60124 Ancona, Italy; 3 Analysis Laboratory, Geriatric Hospital, IRCCS INRCA, 60124 Ancona, Italy; 4 School of Science and Technology, University of Camerino, 62032 Camerino, MC Italy; 5 Rheumatology Division, Geriatric Hospital, IRCCS INRCA, 63900 Fermo, Italy

**Keywords:** Osteoporosis, Fracture risk, Vertebral fracture, Trabecular bone microarchitecture, Magnetic resonance imaging, Fractal lacunarity, Bone mineral density

## Abstract

**Background:**

Osteoporotic fractures are a major cause of morbidity in the elderly. Menopausal women represent the population with the highest risk of early osteoporosis onset, often accompanied by vertebral fractures (VF). Bone mineral density (BMD) is commonly assessed by dual-energy X-ray absorptiometry (DXA) for osteoporosis diagnosis; however, BMD alone does not represent a significant predictor of fracture risk. Bone microarchitecture, instead, arises as a determinant of bone fragility independent of BMD. High-resolution magnetic resonance imaging (MRI) is an effective noninvasive/nonionizing tool for in vivo characterisation of trabecular bone microarchitecture (TBA). We have previously set up an MRI method able to characterise TBA changes in aging and osteoporosis by one parameter, trabecular bone lacunarity parameter β (TBLβ). Fractal lacunarity was used for TBA texture analysis as it describes discontinuity of bone network and size of bone marrow spaces, changes of which increase the risk of bone fracture. This study aims to assess the potential of TBLβ method as a tool for osteoporotic fracture risk.

**Methods:**

An observational, cross-sectional, and prospective study on over-50s women at risk for VF was designed. TBLβ, our index of osteoporotic fracture risk, is the main outcome measure. It was calculated on lumbar vertebra axial images, acquired by 1.5 T MRI spin-echo technique, from 279 osteopenic/osteoporotic women with/without prior VF. Diagnostic power of TBLβ method, by Receiver Operating Characteristics (ROC) curve and other diagnostic accuracy measurements were compared with lumbar spine DXA-BMD.

**Results:**

Baseline results show that TBLβ is able to discriminate patients with/without prevalent VF (*p* = 0.003). AUC (area under the curve from ROC) is 0.63 for TBLβ, statistically higher (*p* = 0.012) than BMD one (0.53). Contribution of TBLβ to prevalent VF is statistically higher (*p* < 0.001) than BMD (sensitivity: 66% vs. 52% respectively; OR: 3.20, *p* < 0.0001 for TBLβ vs. 1.31, *p* = 0.297 for BMD). Preliminary 1-year prospective results suggest that TBA contribution to incident VF is even higher (sensitivity: 73% for TBLβ vs. 55% for BMD; RR: 3.00, *p* = 0.002 for TBLβ vs. 1.31, *p* = 0.380 for BMD).

**Conclusion:**

Results from this study further highlight the usefulness of TBLβ as a biomarker of TBA degeneration and an index of osteoporotic fracture risk.

## Background

Osteoporosis, as defined by World Health Organization (WHO), is a systemic skeleton disease characterised by low bone mass and microarchitectural deterioration of bone tissue with consequent increase of bone fragility and susceptibility to fracture [1]. This pathology can be induced by a variety of causes and has been classified in either primary or secondary osteoporosis. Primary osteoporosis is strictly linked to age-related deterioration of bone tissue while secondary osteoporosis can be the consequence of various conditions and diseases or can be induced by medications that adversely affect bone health. Primary or primitive osteoporosis has been further classified in type I (postmenopausal) and type II (senile) osteoporosis. Type I osteoporosis typically affects 50–65 years old women due to accelerated trabecular bone resorption linked to oestrogen deficiency. Type II (senile) osteoporosis is peculiar to over-65 s subjects when cortical bone deterioration is also involved. The fracture pattern in postmenopausal osteoporosis mainly involves the spine while senile osteoporosis is characterised by fractures mainly affecting hip and femur [2].

Osteoporotic fractures are a major cause of morbidity in the elderly and menopausal women represent the population with the highest risk of early osteoporosis onset. The increased lifespan in the industrialized world accounts for increasing incidence of osteoporosis and bone fractures with a perspective of additional years (at least 20) of disability osteoporotic women have to face in their later life [3, 4]. The dimension and complexity of bone fragility problem are huge: in Europe, osteoporosis causes every year almost 1 million hip fractures, 500,000 femur fractures, and 1,400,000 vertebral fractures (VF) over those to other sites (wrist, humerus, ribs). Hip fractures (75%) and VF (85%) mainly affect women. The remaining lifetime probability in women at the menopause of a fracture at any site exceeds that of breast cancer (approximately 12%). The likelihood of a fracture at any of these items is 40% or more in developed countries, a picture close to the probability of coronary heart disease [4–6].

Due to the silent progression of bone structure degeneration, osteoporosis diagnosis often follows a painful fracture event. Currently, only a small percentage of individuals knows to be osteoporotic while the condition of most pathologic people remains undiagnosed until a fracture occurs [7]. VF may cause acute pain and loss of function, but may also occur without serious symptoms; however, they often recur, and the consequent disability increases with the number of fractures. It is worth noting that the first fracture event increases the risk and accelerates the onset of new ones [8].

The estimate of areal bone mineral density (BMD) by means of dual-energy x-ray absorptiometry (DXA) represents the most common approach to diagnose osteoporosis and predict fracture risk. However, BMD alone is not a good predictor for fracture risk [9, 10]. Over the past decades, it has been recognized that factors of bone quality also contribute to fracture risk. In particular, microarchitecture emerges as a determinant of bone strength independent of BMD [11, 12] and its investigation would give insight into the mechanisms of bone fragility as well as the action of drugs used to prevent osteoporotic fractures [11].

The rapid spreading of medical imaging techniques in clinical practice, together with the impressive development of information technologies, has solicited proliferation of new methods for in vivo assessment of trabecular bone microarchitecture (TBA) changes with ageing and osteoporosis [13, 14]. Developments in high-resolution magnetic resonance imaging (MRI) techniques have expanded perspectives for in vivo characterisation of TBA by noninvasive/nonionizing methods [15]. Nevertheless, texture analysis, mainly based on classic histomorphometric methods, is not frequently used in clinical practice because of the large number of calculated parameters that makes difficult their interpretation.

An original and innovative method of MR image analysis, developed in our Institute, has been previously proposed to provide a unique parameter sensitive to TBA changes in ageing and osteoporosis [16, 17]. It has been set up by considering the complexity of human beings and fractal properties of several anatomic and physiologic structures among which is bone tissue [14, 18–23]. Characterising TBA by fractal lacunarity seems to be a suitable approach. Fractal lacunarity, in fact, by measuring space-filling capacity of a complex object, has the potential to describe both bone network discontinuity and sizes of trabecular spaces (bone marrow) [17, 24], changes of which represent an index of increased fracture risk. The mathematical solution proposed is one parameter calculated from the simple hyperbola formula that well fits the curvilinear plot obtained from lacunarity analysis of trabecular network [14, 16, 17, 25]. In previous studies, we observed that parameter β, representative of lacunarity, correlates with both age and physio-pathologic status [14, 16, 17, 25]. Therefore, parameter β (namely TBLβ: trabecular bone lacunarity parameter β) is the natural candidate to become a standard for TBA characterisation and a potential index of bone fragility fracture risk.

Here we present an observational, cross-sectional, and prospective study on over-50s women at risk for osteoporotic fractures designed for diagnostic power assessment of this potential new diagnostic tool. In particular, baseline results from LOTO (Lacunarity Of Trabecular bone in Osteoporosis) study are described and discussed. Preliminary 1-year prospective results are also presented.

## Methods

### Study design and participants

An observational, cross-sectional, and prospective study was designed to assess the diagnostic power of the new tool, potentially useful for early diagnosis of fracture risk in osteoporotic pathology. It is based on fractal lacunarity analysis of TBA in MRI lumbar vertebra images.

Osteopenic/osteoporotic over-50s women, at risk for bone fragility-spontaneous fractures, were recruited for baseline assessment as described below. Follow up at 1, 2 and 5 years have been also planned.

The main objective of the present study is to verify the potential of TBLβ in discriminating patients with and without bone fragility VF by assessing diagnostic power of TBLβ method in baseline data. The main outcome measure is TBLβ of our bio-mathematical model as an index of osteoporotic fracture risk. It was calculated by means of a software prototype, developed by adopting a greyscale version of the method [14] outlined below, on L4 axial section images acquired by 1.5 T MRI spin-echo multislice technique. DXA-BMD at L1-L4 lumbar spine was used as the reference standard.

### Ethical standards

The LOTO study was approved by the institutional Ethical Committee (FiORdiLOTO SC/11/281) and was performed in accordance with the Declaration of Helsinki (1964) and its later amendments.

Written informed consent was received from participants prior to inclusion in the study.

### Procedures

After approval by the Ethics Committee of our Institute, over-50s women defined osteopenic/osteoporotic on the bases of DXA T-score at L1-L4 lumbar spine were enrolled among those who asked for our Institute BMD-Service based on the following inclusion/exclusion criteria.

Patients were enrolled when eligible for all the following characteristics and conditions: consecutive patients who accepted to be clinically followed by our Bone Metabolism Unit; age of 50 years and older; densitometric diagnosis of osteopenia (T-score between − 1 and − 2.5) or osteoporosis (T-score equal to − 2.5 or lower) at L1-L4 lumbar spine; primitive (postmenopausal and senile) osteoporosis, with or without prior vertebra bone fragility fractures; have given written informed consent.

Patients were excluded when at least one of the following conditions or pathologies was present: osteoporosis secondary to drug-induced bone loss, chronic diseases, or genetic diseases; contraindications to MRI; severe impairment of cognitive/functional status.

After first visit and interview to record demographic and clinical data, patients underwent the regular diagnostic practice for osteoporosis and osteoporotic fractures (DXA, blood analysis, dorsal-lumbar spine x-ray). Patients characterised as described above underwent lumbar spine MRI to acquire spin-echo axial multislice vertebral images for TBA characterisation.

### Bone mineral density assessment

BMD measurement to classify patients in terms of osteopenia/osteoporosis was performed by means of a DXA fan bean system (low x-ray emission) (Lunar Prodigy Primo - GE Medical Systems). Densitometry reports were based on WHO criteria for DXA lumbar and femoral scans by expressing results as T-score and Z-score. In particular, based on T-score values at L1-L4 lumbar spine, patients’ BMD was defined as: normal (T-score ≥ − 1 SD); low bone mass (osteopenia) (T-score between − 1 and − 2.5 SD); osteoporosis (T-score ≤ − 2.5 SD). Z-score, reflecting bone density compared with other people in the same age-group and of the same size and gender, was considered to exclude secondary osteoporosis. In fact, if this score is unusually high or low, it may indicate that factors other than age can affect BMD.

### X-ray of dorsal-lumbar spine

A conventional x-ray apparatus with remote control (Prestige-GE Medical Systems) was used to acquire images of dorsal-lumbar spine to be interpreted by morphometry techniques for the diagnosis of VF. Exposure technique of spine in lateral projection was applied according to Genant criteria [26] for morphometry analysis. A computed radiography system with morphometry software for visualization/analysis (Medstation-Exprivia) was used and the quantitative method was applied. It allows fracture diagnosis based on both reduction of 4 mm threshold value and 15% reduction of at least one height of vertebral body. Morphometry on conventional x-ray images (MRX) has been preferred to that on densitometric images (MXA) as it represents the best approach for the diagnosis of osteoporotic fracture prevalence. MRX allows obtaining both semiquantitative and quantitative evaluation with higher precision than MXA [26].

### Magnetic resonance imaging of lumbar spine

High resolution MRI, 1.5 T whole body system (Gyroscan Intera; Philips-Medical System, ACR-Nema 1.0), was used for dorsal-lumbar spine MRI scan using a phased array dS Spine coil. Spin-echo multislice technique was applied for the acquisition of TBA axial section images of vertebral bodies (9 to 12 slices with a thickness of 3 mm without space gap between slices). The pulse sequence was as follow: TE of 15 ms, TR of 525 ms; flip angle was of 90°, matrix of 512 × 512, and pixel size equal to 0.469 mm for a scan time below 15 min. To confirm the reproducibility of this image acquisition method assessed in previous studies, the pulse sequence was initially tested on two subjects undergone lumbar spine MRI scan for three times in two weeks. As previously reported [14, 16, 17], the method was set up on real images to avoid failure that often occurs when phantom and/or simulation are used to develop new methods. Spin-echo technique was systematically applied to L1-L4 lumbar spine.

### Estimate of TBLβ in magnetic resonance images

Computation of TBLβ, potential index of fracture risk, was performed on MR spin-echo images of lumbar vertebras by adopting a method previously developed in our laboratory as described in [16, 17] and modified in [14, 25].

Briefly, to estimate lacunarity we chose the gliding box algorithm, GBA, based on the analysis of mass (M) distribution in binary images [27]. A simple extension of this algorithm was used to deal with greyscale images. In this extension, the moment formula for the discrete distribution of M was upgraded with the moment formula for M taking a continuous range of possible values, see [14, 25] for details. The efficiency of such an extension was improved by a different pre-processing step through a sigmoid function to weight grey level of each pixel.

The GBA method was implemented in software, by using MATLAB software package (the MatWorks, Inc.), to operate on both binary images, after a pre-processing step involving greyscale reversion (version 1) [16, 17] and on original greyscale images with and without reversed greyscale (versions 2 and 3) [14, 25]. In these versions, a sigmoid function is used for grey levels’ rescaling. This procedure allows weighting grey level for each pixel, thus limiting the risk of information loss due to image binarization. Version 2 was considered in the present study, as it is more sensitive than version 1 and more robust than version 3 [14].

**Fig. 1:**
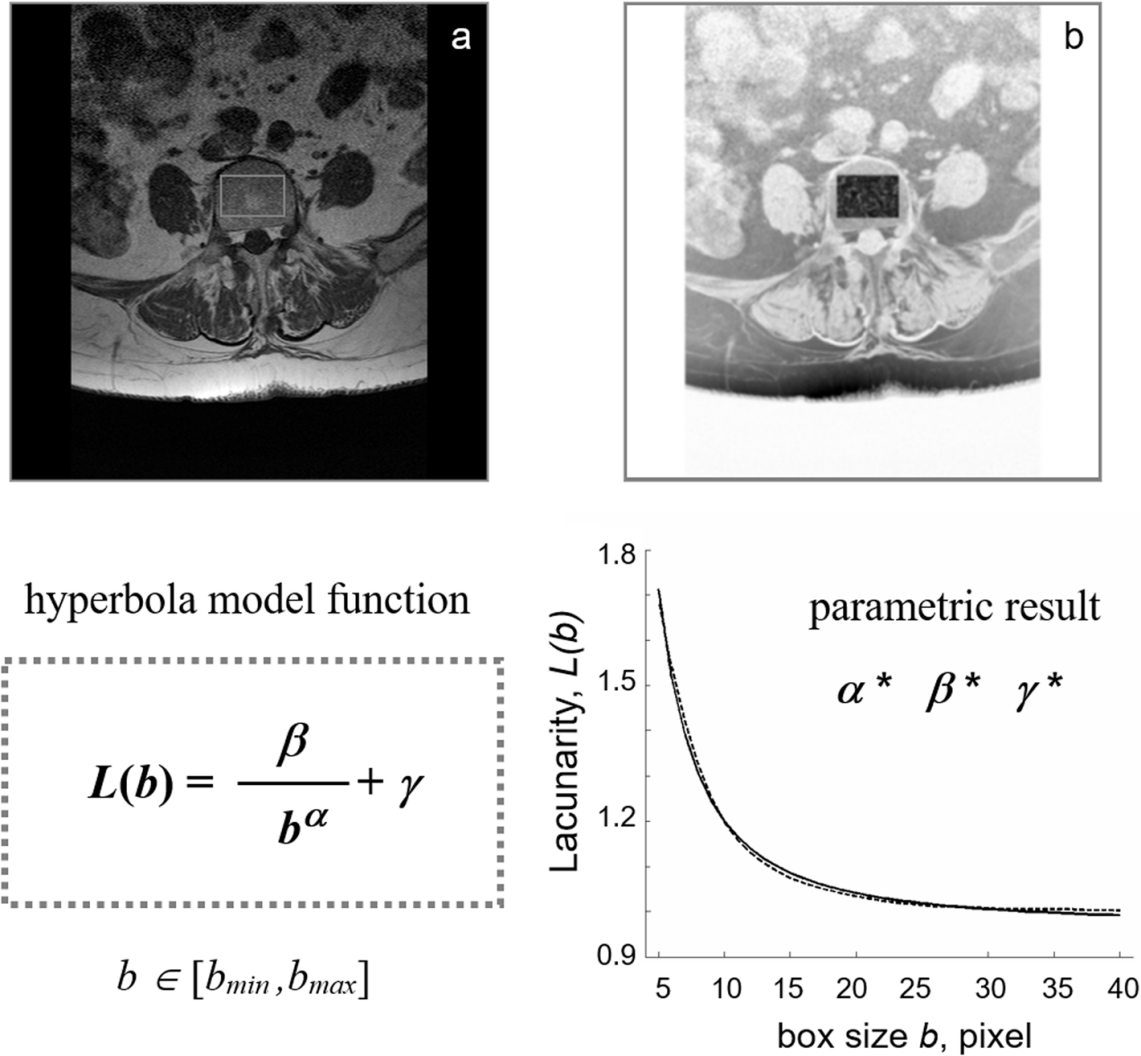
Schematic representation of TBLβ method. **a** 1.5 T MRI spin-echo image (512 × 512 pixel, pixel size equal to 0.469 mm) of fifth out of nine axial section of the 4th lumbar vertebra; **b** Rectangular ROI within the inner perimeter of vertebral body in an intermediate step of image processing on reverse greyscale image. The plot (right bottom) represents the result of gliding box algorithm application (dotted line) as fitted by hyperbola model function (solid line) used to calculate the triplet of parameters α*, β*, γ* (left bottom). TBLβ: trabecular bone lacunarity parameter β; MRI: magnetic resonance imaging; ROI: region of interest

Figure 1 shows a schematic representation of the method. In particular, the following model:

$$ L(b)=\frac{\beta }{b^{\alpha }}+\gamma, \kern0.36em b\in \left[{b}_{\mathrm{min}},{b}_{\mathrm{max}}\right] $$

was chosen to approximate the hyperbola-like curvilinear plot of lacunarity function L(b) where α, β, γ are suitable parameters [16, 17]. Dealing with fractals, α is related to the fractal dimension of the set and β (our TBLβ) characterises the lacunarity of the set [14, 27]. Low TBLβ values correspond to high lacunarity, that is, high deterioration of TBA that predisposes to increased fracture risk.

The standardized version of our method was set up by using the middle axial section(s) of L4. Lacunarity analysis on the other sections of the same vertebra and on other vertebras (i.e. L2) was performed to further validate the standardized version or to suggest further improvements. Intra- and inter-operator variability was confirmed always lower than 2% by randomly repeating twice ROI selection and lacunarity estimate.

### Sample size and statistical analyses

Taking into account the primary objectives of LOTO study, that is, diagnostic capacity and accuracy of TBLβ method, the sample size was estimated of 280 osteopenic/osteoporotic patients, 224 without VF and 56 with prior VF based on a 20% prevalence of VF in over-50s women, a non-relevant incidence in two-year follow up, and a 10% drop out. Sample size was calculated based on: 0.05 first type error; more than 80% study power; AUC (area under the curve) in ROC (receiver operating characteristics) curve analysis for diagnostic accuracy measurement; 0.80 high level of diagnostic accuracy; 0.10 moderate difference between TBLβ and DXA-BMD; 1/4 patient ratio with/without VF; a moderate variability among observers.

Statistical analyses were performed by means of SPSS package v. 19 (SPSS Inc. Chicago IL) and statistical significance was accepted for *p* ≤ 0.05. Descriptive statistics were used to synthesize demographic and clinical-pathologic characteristics of the whole patients’ sample. Univariate analysis was performed for continuous variables. Student’s *t*-test and Chi-squared test were used to compare differences between groups. Nonparametric alternative Mann-Withney U test was used when normality assumption, checked by the Kolmogorov-Smirnov test, was not valid. Diagnostic accuracy of TBLβ method in predicting VF was evaluated by ROC curve analysis (AUC) that provides a combined measure of sensitivity (SN) and specificity (SP). Statistical significance of discriminating power of the test was defined by zeta test. The best cut-off values of TBLβ to predict VF, defined by Youden index in ROC curve and by median value from the whole sample, were used to calculate also other diagnostic accuracy measurements (odds ratio, OR; relative risk, RR and attributable risk, AR; SN, SP, positive and negative predictive values (PPV and NPV), and effectiveness (accuracy, ACC).

## Results

### Demographic and clinical characteristics

**Fig. 2:**
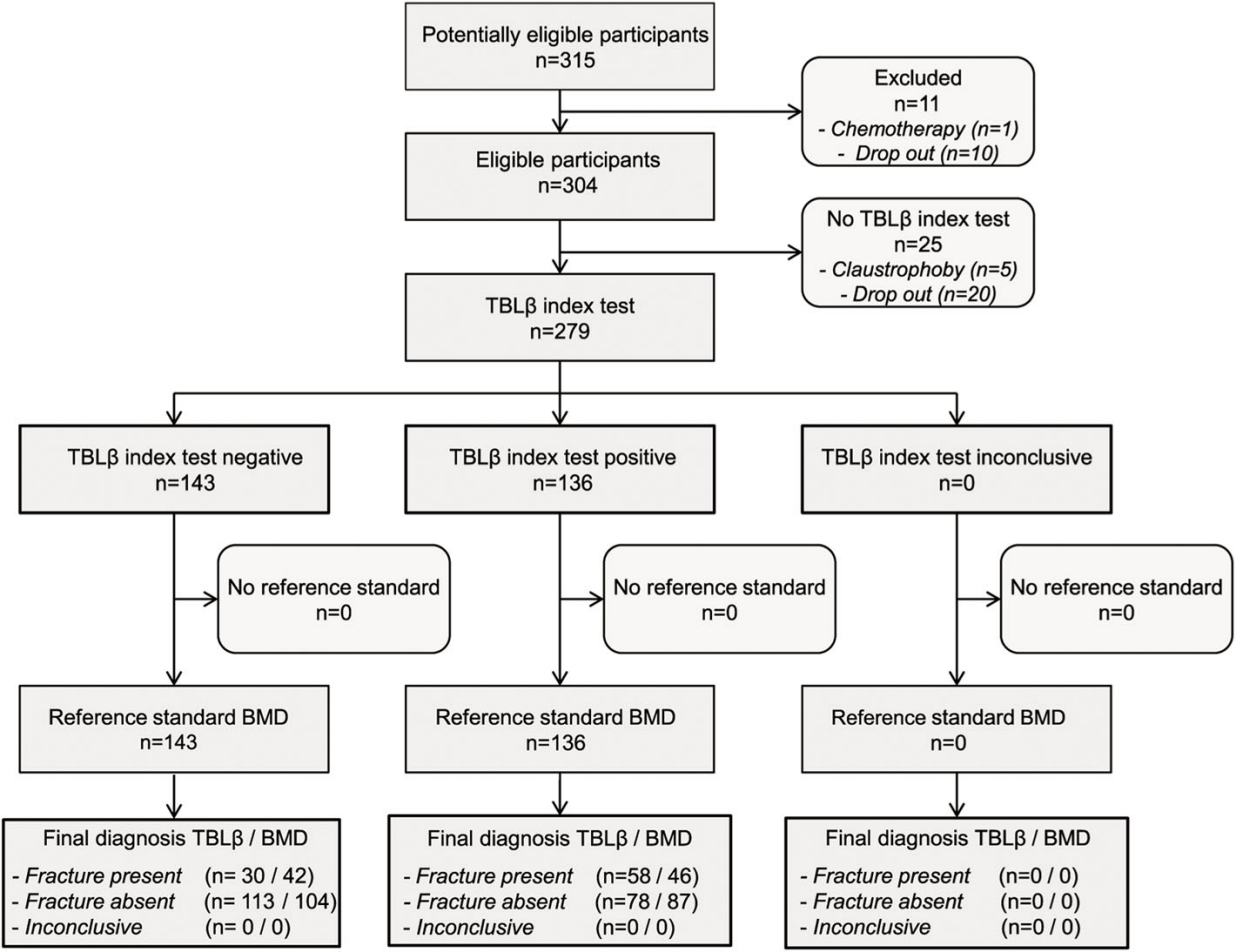
Flow chart of LOTO study to assess diagnostic accuracy of TBLβ vs. BMD. TBLβ: trabecular bone lacunarity parameter β; BMD: bone mineral density

A complete dataset of baseline recording was obtained for 279 out of 315 subjects eligible for the study (Fig. 2). Table 1 summarizes main demographic and clinical characteristics of the whole sample and of the two subgroups considered: VF+, with prevalent VF (n 88, 32%) and VF-, without VF (n 191, 68%).

**Table 1:** Demographic and clinical characteristics of LOTO patients

Characteristic	Overall	without VF	with VF
	*N* = 279	*N* = 191	*N* = 88
Age, years	60 ± 7	59 ± 7	62 ± 7
< 65	212 (76.0)	153 (80.1)	59 (67.0)
≥ 65	67 (24.0)	38 (19.9)	29 (33.0)
Body mass index, kg/m^2^	23.09 ± 3.00	22.98 ± 2.94	23.32 ± 3.14
≤ 25	217 (77.8)	152 (79.6)	65 (73.9)
> 25	62 (22.2)	39 (20.4)	23 (26.1)
Bone mineral density, L1-L4 T-score	−2.4 ± 0.9	− 2.4 ± 0.9	− 2.5 ± 0.8
≤ − 2.5	133 (47.7)	87 (45.5)	46 (52.3)
> − 2.5	146 (52.3)	104 (54.5)	42 (47.7)
Osteoporosis medication			
any medication	123 (44.1)	70 (36.6)	53 (60.2)
Vitamin D and/or Calcium supplements ^a^	95 (77.2)	55 (78.6)	40 (75.5)
Bisphosphonates ^a^	70 (56.9)	43 (61.4)	27 (50.9)
other ^a^	10 (8.1)	3 (4.3)	7 (1.3)

Data are represented as mean ± SD or n (%). VF: prevalent vertebral fracture

^a^ Percentage calculated within treated patients’ group

The age range of the whole sample was 50–85 years (mean age ± SD equal to 60 ± 7, median 59). A similar age distribution was observed in VF- (mean age 59 ± 7 years, range 50–85, median 58) while VF+ showed a statistically higher mean age (62 ± 7 years, range 51–80, median 62, *p* = 0.001) when compared with VF-.

**Fig. 3:**
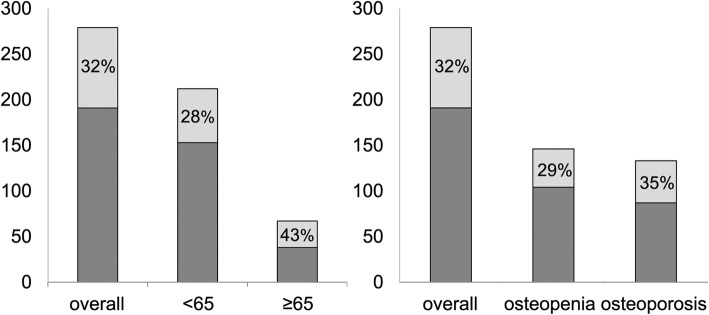
Prevalence of bone fragility vertebral fractures (light grey) in osteopenic/osteoporotic over-50s women related to both age (left) and BMD (right). BMD: bone mineral density

BMD T-score at L1-L4 lumbar spine was found equal or lower than − 2.5 SD (osteoporosis) in 47.7% women. Prevalent VF were found in 31.5% subjects, 47.7% of which defined osteopenic at lumbar spine by DXA-BMD and 67% younger than 65 years. Figure 3 shows the prevalence of VF within both age and BMD subgroups.

The last, but not least, discrimination regarding the sample under study deals with osteoporosis treatment. We found that 123 out of 279 patients (44%) were with at least one osteoporosis medication at baseline, 77% of which with Vitamin D and/or Calcium supplements and 57% under bisphosphonates treatment (with or without Vitamin D/Calcium supplements), while other osteoporosis medications accounted for only 8%. Among overall treated (T+) subjects, 57% were VF- (mean age 62 ± 7 years; range 51–85) and 43% were VF+ (63 ± 8 years; range 51–80) while untreated (T-) subjects accounted for 78% VF- (mean age 58 ± 6 years; range 50–81) and 22% VF+ (mean age 61 ± 7 years; range 51–74).

### TBLβ as an index of osteoporotic fracture risk

**Fig. 4:**
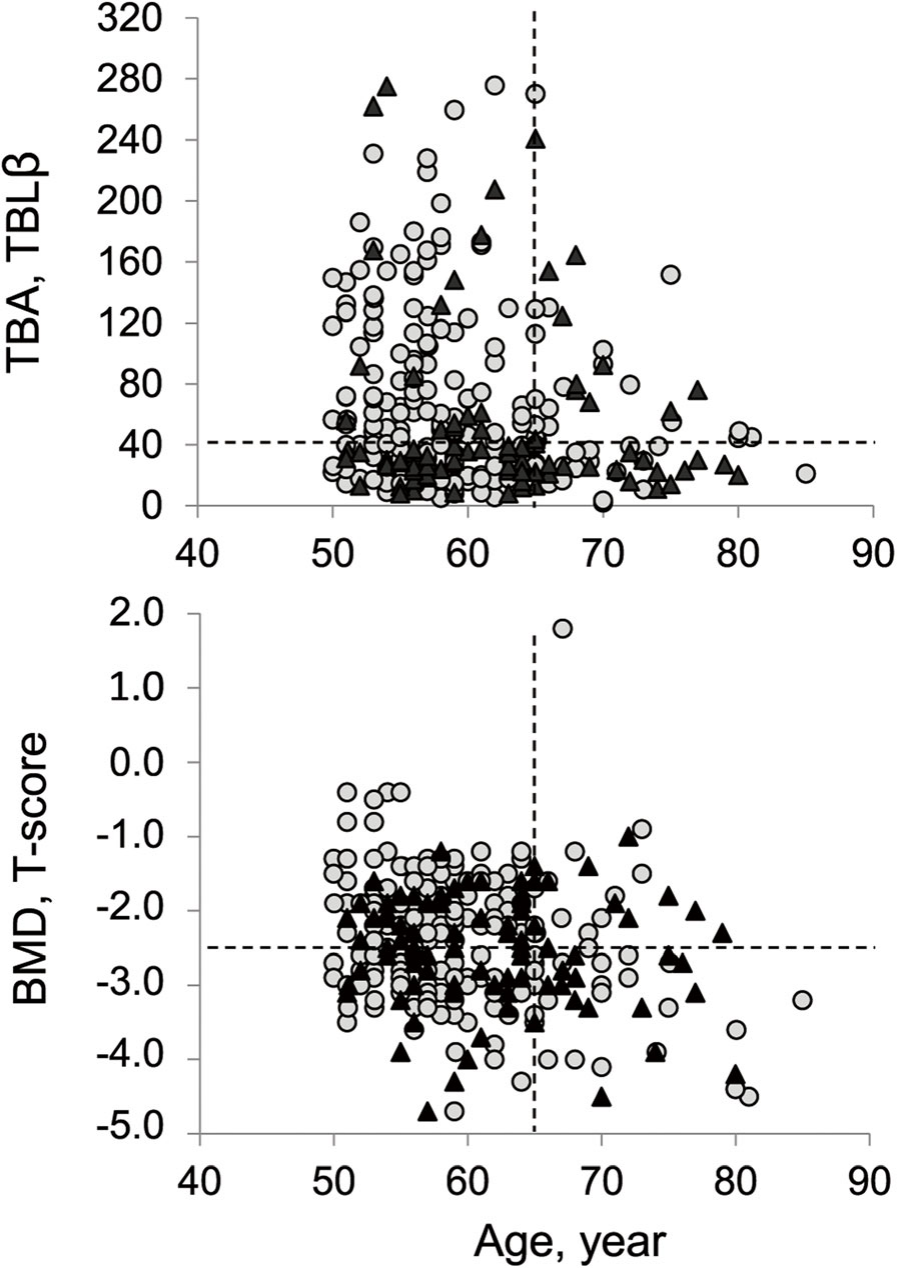
Age-related distribution of TBLβ and BMD in osteopenic/osteoporotic over-50s women. TBLβ (top) at a cut-off equal to 40 is able to separate women with (black triangle) and without (grey circle) vertebral fracture better than BMD (bottom) at a cut-off value of T-score equal to − 2.5. TBLβ: trabecular bone lacunarity parameter β; BMD: bone mineral density

TBLβ was calculated as the mean value of the two central L4 axial sections (i.e. 5th and 6th of 10). Figure 4 shows age-related distribution of TBLβ and lumbar spine BMD in VF+ and VF- subjects. TBLβ values were not normally distributed; therefore, results in Table 2 were expressed as median (interquartile range) and *p* values were estimated by Mann-Whitney U test. Results from the whole sample showed that TBLβ is able to discriminate between VF+ and VF- with values statistically lower (*p* = 0.002) in VF+ when compared with VF- independently on BMD. In fact, osteoporotic and osteopenic patients did not show any statistical difference (*p* = 0.795). A statistically significant difference (*p* = 0.001) exists between VF+ and VF- within the osteoporotic group but not within osteopenic one despite a lower value in VF+ osteopenic patients when compared with VF- ones.

**Table 2:** TBLβ vs. BMD in over-50s women with/without bone fragility vertebral fracture

Subjects	TBLβ		*P*	BMD		*P*
	VF-	VF+		VF-	VF+	
Overall	51 (28–101)	31 (23–60)	0.002	0.895 ± 0.107	0.875 ± 0.094	0.131
Osteopenic	47 (23–104)	38 (24–89)	0.123	0.953 ± 0.040	0.972 ± 0.071	0.071
Osteoporotic	52 (32–93)	30 (23–51)	0.001	0.803 ± 0.057	0.804 ± 0.069	0.351
Untreated	52 (29–107)	30 (23–53)	0.027	0.920 ± 0.106	0.885 ± 0.096	0.039
Treated	50 (26–93)	31 (23–62)	0.078	0.853 ± 0.095	1.025 ± 1.148	0.280

Values are median (interquartile range) for TBLβ and mean ± standard deviation for BMD

TBLβ: trabecular bone lacunarity parameter β; BMD: bone mineral density expressed in mg/cm^2^; VF+: with vertebral fracture; VF-: without vertebral fracture; P: Statistical significance for *p* ≤ 0.05

### Predicting bone fragility vertebral fractures by TBA and BMD

**Fig. 5:**
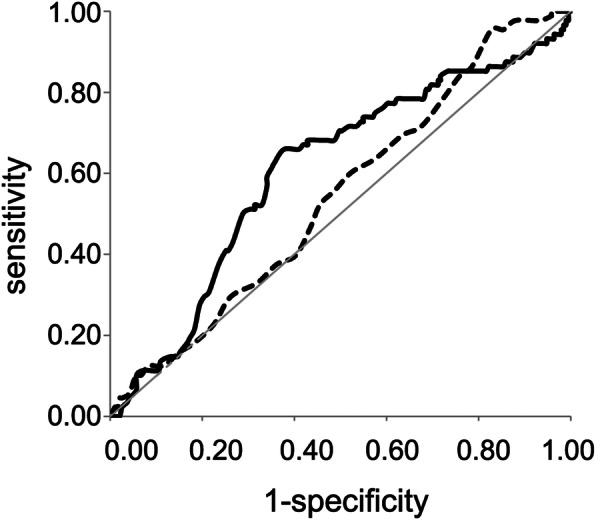
Empirical ROC curves for both TBLβ (solid line) and BMD T-score (dotted line) data sets. Light grey line represents the reference line. Each point in the curves represents true positive (sensitivity, ordinate axis) vs. false positive (1-specificity, abscissa axis) for different thresholds. ROC: receiver operating characteristics; TBLβ: trabecular bone lacunarity parameter β; BMD: bone mineral density

Comparison of ROC curves for TBLβ and BMD T-score is illustrated in Fig. 5. Here we report results related to TBLβ cut-off value 40 (mean value between Youden index: 39 and median value from the whole sample: 41). The same cut-off value was previously observed in a pilot study. By ROC curve analysis, AUC for TBLβ was 0.63 (z = 3.795; *p* = 0.005), statistically higher (*p* = 0.032) than BMD T-score (threshold = − 2.5 SD) with an AUC equal to 0.53 (z = 2.400; *p* = 0.016). It is worth noting that by using BMD expressed as g/cm^2^ we obtained comparable results (data not shown). Sensitivity, specificity, positive predictive value (precision), negative predictive value and accuracy (effectiveness) were estimated for TBLβ vs. BMD respectively as follow: SN = 0.66 vs. 0.52; SP = 0.59 vs. 0.54; PPV = 0.43 vs. 0.35; NPV = 0.79 vs. 0.71, ACC = 0.64 vs. 0.54.

**Fig. 6:**
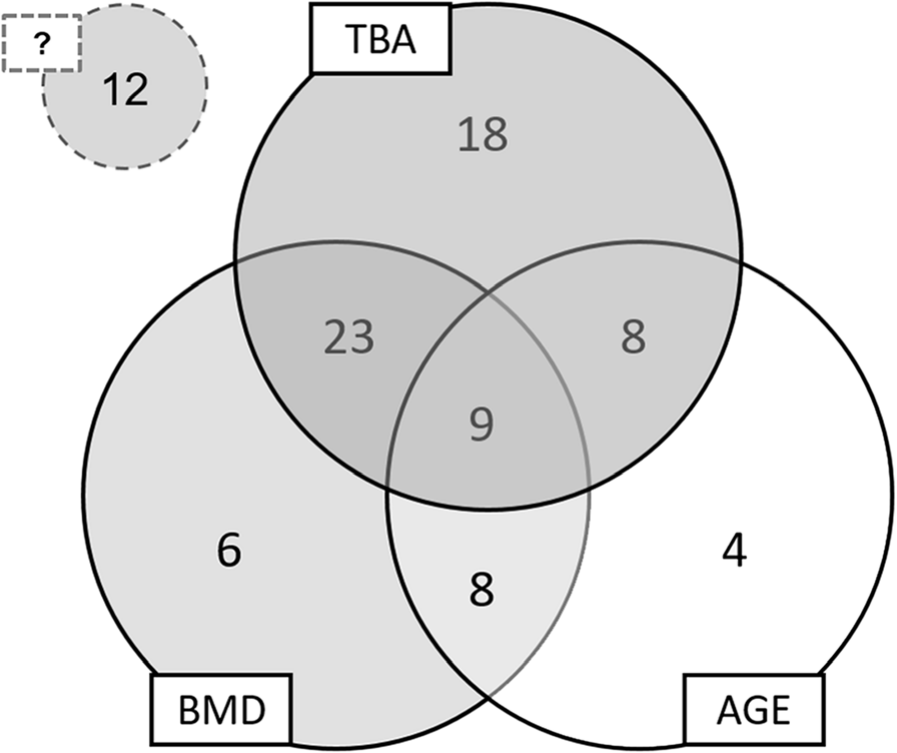
Venn diagram of osteoporotic fracture risk factors for prevalent vertebral fractures in over-50s women. Contribution of TBA (TBLβ≤40, 66%) alone is higher than AGE (years≥65, 33%) and BMD (T-score ≤ − 2.5, 52%). Combining the three risk factors would contribute to identify 86% patients at high risk of osteoporotic fractures. Other risk factors (?) are responsible for prevalent fractures in 14% women. TBA: trabecular bone microarchitecture; TBLβ: trabecular bone lacunarity parameter β; BMD: bone mineral density

OR calculated in the whole sample according to Altman [28] was equal to 2.80 for TBLβ (95% CI: 1.6359 to 4.7433; *p* < 0.001) corresponding to a statistically significant moderate association of TBLβ with prevalent VF while BMD T-score showed a weak association (OR = 1.31; 95% CI: 0.7893 to 2.1717; *p* = 0.297). Venn diagram (Fig. 6) of osteoporotic fracture risk factors for prevalent VF in over-50s women showed that the contribution of TBA (TBLβ ≤40, n. 58 out of 88, 66%) alone is higher than age (years ≥65, n. 29, 33%) and BMD (T-score ≤ − 2.5, n. 46, 52%). Combining the three risk factors would contribute to identify 86% patients at high risk of osteoporotic VF. Other risk factors responsible for prevalent VF account for 14%.

We observed that VF+ subjects with TBLβ > 40 included several T+ patients. Therefore, to exclude the influence of any medication on both TBA and BMD, we considered only T- subjects. We found that, among 156 T- patients, 69% (24 out of 35) were associated with a TBLβ ≤40 vs. 49% patients (17 out of 35) with a BMD T-score ≤ − 2.5 (Table 3). OR was 3.10 (95% CI: 1.3919 to 6.8962; *p* = 0.006) for TBLβ and 1.49 (95% CI: 0.6975 to 3.1701; *p* = 0.304) for BMD T-score, that account for a statistically significant moderate association of TBLβ with prevalent VF against a weak not significant association for BMD T-score.

**Table 3:** Contribution of TBA and BMD to prevalent and incident bone fragility vertebral fractures

	marker	prevalent VF		incident VF^a^	
		overall	T-	overall	T-
number (VF+/VF-)		88/191	35/121	33/122	18/62
TBA	TBLβ ≤40	66%	69%	73%	78%
BMD	T-score ≤ −2.5	52%	49%	55%	50%

TBLβ: trabecular bone microarchitecture parameter β; BMD: bone mineral density; VF: vertebral fracture; VF+: with VF; VF-: without VF; T-: untreated at baseline

^a^ Preliminary results from 1-year follow up

### Preliminary 1-year prospective results

We observed that several VF- patients had a TBLβ ≤40, that is, at risk for bone fragility fracture. Preliminary results on incident VF (Table 3) showed that, among 155 patients at 1-year follow up, 73% incident VF+ subjects (24 out of 33) were associated with a TBLβ ≤40 at baseline vs. 55% (18 out of 33) for BMD T-score ≤ − 2.5. The relative risk (RR) calculated according to Altman [28] showed a statistically significant moderate association of TBLβ with incident VF (RR = 3.00, 95% CI: 1.4902 to 6.0210; *p* = 0.002) against a low association for BMD T-score (RR = 1.31; 95% CI: 0.7148 to 2.4136; *p* = 0.380). It is worth noting that in 80 T- patients at baseline, 78% incident VF+ subjects (14 out of 18) were associated to a baseline TBLβ value ≤40 vs. 50% for BMD (9 out of 18). The related RR = 3.17 (95% CI: 1.1409 to 8.7897; *p* = 0.027) indicates a moderate association with incident VF against a weak association for BMD T-score (RR = 1.29; 95% CI: 0.5711 to 2.8946; *p* = 0.544). Results from AR calculation in the whole sample suggest that by monitoring TBA degeneration as a risk factor for bone fragility fracture would reduce VF incidence of about 25% (AR = 0.24) against only 6% in the case of BMD (AR = 0.06).

## Discussion

In this study, we present a new potential diagnostic tool useful in the management of fracture risk in osteoporosis pathology. An observational, cross-sectional, and prospective study (LOTO) on osteopenic/osteoporotic over-50s women, at risk for bone fragility fracture, was designed for diagnostic assessment of the method. It is based on fractal lacunarity texture analysis of TBA in lumbar vertebra images acquired by 1.5 T MRI system [14, 17, 25]. The method provides only one parameter, TBLβ, particularly sensitive to TBA degeneration [14]. Baseline results from LOTO study show that TBLβ is statistically lower in subjects with prevalent VF than in non-fractured ones. In addition, TBLβ is able to discriminate between subjects with and without VF better than BMD thus becoming an index candidate of osteoporotic fracture risk assessment.

Currently, DXA is commonly used to measure BMD and predict fracture risk. However, reliable BMD prediction of osteoporotic fractures is obfuscated by the significant overlap between subjects with and without bone fragility fractures [29]. In this context, criteria recommended by WHO for osteoporosis diagnosis and related therapeutic treatments to care for the disease and prevent bone fracture deserve some discussion [3, 30, 31] mainly as far as age and BMD as the risk factors are concerned. In Italy, as in many other industrialized countries, BMD measurement has been for long time mandatory only after 65 years of age in the absence of other risk factors [32]; nevertheless, most menopausal women do not perceive the severity of this pathology until a fracture event occurs due to the silent onset and progression of osteoporosis.

Results from LOTO study on the prevalence of bone fragility VF in relation to the two main risk factors (age ≥ 65 and BMD T-score ≤ − 2.5 as indicated by WHO), for primitive osteoporosis [3, 30–32] and consequent bone fragility fractures, confirm literature evidence [3, 5, 29, 31]. In particular, a relevant percentage of VF has been found in women younger than 65 years. It is worth noting that a high probability exists for women with menopausal osteoporotic VF to experience a senile osteoporotic femur fracture. On the other hand, lowering age limit is not sufficient to improve the management of primitive postmenopausal osteoporosis. In fact, BMD alone is not a good predictor of fracture risk [10] as confirmed in this study: 29% of osteopenic women have already experienced a vertebral bone fragility fracture that account for more than 50% prevalent fractures in LOTO patients.

Screening of the population at risk for bone fragility and treatment assessment of patients to prevent consequent fractures would be useful tools to increase quality of life in the elderly and to lighten the related healthcare-socio-economic impact. Noninvasive tools are necessary to characterise bone quality beyond bone mass to accurately assess the individual risk of bone fragility fracture and to evaluate the progression of osteoporosis and the efficacy of its treatment [10, 32, 33].. WHO recommended osteoporosis diagnosis be made a DXA-BMD measurement when noninvasive technologies to assess bone structure in vivo were not available yet. It is worth noting that guidelines for osteoporosis management have been already updated by introducing bone quality evaluation [32, 34, 35]. Nevertheless, BMD and age remain the main risk factors to be considered for bone fragility fracture risk. The limits of these two fracture risks can be overcome by adopting algorithms, i.e. FRAX, to better predict fracture risk and decide for osteoporosis treatment [36, 37]. FRAX, a fracture risk assessment tool, estimates the 10-year probability of hip and major osteoporotic fractures based on the individual’s risk factor profile [36]. However, it has been emerging that FRAX does not add improvement in fracture risk assessment when compared to BMD in peri- and early postmenopausal women [38, 39]. DXA devices of last generation have been starting to be equipped with dedicated software for bone quality investigation. Nevertheless, this kind of technology, despite the name (TBS: trabecular bone score), cannot investigate TBA [40, 41].

The introduction of nonionizing/noninvasive tools to quantify TBA in clinical practice would complete the diagnosis of osteoporosis as defined by WHO [1]. MRI-based diagnosis could complement standard BMD methods for assessing osteoporosis and monitoring longitudinal changes. MRI, in fact, has been emerging as a useful tool in the study of osteoporosis. Several aspects of this technology candidate MRI as a noninvasive/nonionizing tool for in vivo study of bone tissue applicable to humans: MRI does not use ionizing radiation, allows direct acquisition of multiplanar images, and can explore bone physiology features otherwise not investigable by other imaging techniques [13]. As a matter of fact, quantitative MRI techniques, from diffusion tensor imaging (i.e. mean diffusivity and fractional anisotropy) to chemical shift imaging (i.e. T2* and quantitative susceptibility mapping), have been used to discriminate benign and malignant compressed vertebras [42, 43]. Among recent MRI methods to analyse bone tissue in osteoporosis [44, 45], most efforts have been devoting to fat fraction quantification at the spine by analysing bone marrow composition (for review see [46]). However, there are not studies on large cohorts to accomplish the transition of these methods into clinical practice, partially because of limited evidence of their usefulness in predicting fracture risk.

As far as TBA characterisation is concerned, comparing CT (computer tomography) with MR imaging, CT has the advantage of visualizing bone with higher spatial resolution, but the disadvantage of applying considerable radiation dose not applicable to the central skeleton. Bone structure measurements by MRI have been found to be similar to histological or micro-CT ones and highly correlated to HR-pQCT (high resolution–peripheral quantitative CT) [47]. TBA parameters as measured by 3.0 T have been found statistically better than 1.5 T MRI when compared with micro-CT as the reference standard. At 3.0 T the effect of magnetic susceptibility, responsible for apparent increase in trabecular thickness [13], is higher than that at 1.5 T [48, 49]. The apparent increase in trabecular thickness, an artefact due to magnetic susceptibility differences between bone and bone marrow, has the advantage to visualize the smallest trabeculas undetectable in normal conditions because of partial volume effect [33]. At present, most researchers use MR 3D or 2D images acquired by ether gradient-echo or spin-echo techniques. This last modality has the advantage to reduce the above-mentioned magnetic susceptibility related artefact [13].

In early studies, MRI parameters of TBA were found to separate patients with and without osteoporotic fractures better than BMD [50]. Traditional morphometric parameters, such as bone volume fraction (BV/TV), trabecular bone number (Tb.N), and thickness (Tb.Th), showed superior results compared to BMD in separating fractured and non-fractured groups [51]. At present, prospective trials on osteoporotic fractures or large-scale therapeutic trials based on MRI-TBA characterisation are rare [52] while most recent studies deal with BMD alone as the main endpoint [53, 54]. However, this kind of studies would prompt up the definition of a diagnostic set of markers able to complement and/or improve fracture risk assessment based on DXA-BMD. The main obstacles to reach this goal are: limited dissemination of technology in healthcare centres, minimum protocols’ standardization for both image acquisition and image processing, and the high number of parameters to characterise TBA. As a matter of fact, one most recent longitudinal study on alendronate treatment used 3.0 T MRI [55]. Image acquisition was related to mirror sites such as distal tibia, distal radius, and proximal femur. BV/TV, Tb. N, Tb. Th, and Tb. Sp (spacing) were among TBA parameters analysed. Seven additional parameters by geodesic topological analysis (GTA) were also included. Apparent Tb. N and four GTA parameters showed statistical treatment effects only in the distal tibia after 24 months when compared to BMD.

New MR imaging modalities, recently proposed for TBA characterization in both early diagnosis and treatment assessment of osteoporosis, have the limitation of using ever increasing powerful instrumentations (3, 7, and even 11 T) for 2D or 3D TBA imaging in peripheral sites and characterisation by classic histomorphometric analyses [14]. This is mainly due to the choice of micro-CT as the reference standard [47, 54] that alienates the application in clinical setting of a promising noninvasive/nonionizing diagnostic tool.

The original and innovative proposed method, based on fractal lacunarity analysis of vertebral TBA, [14, 16, 17, 21, 25] appears particularly promising. It uses 1.5 T MRI widely available in most healthcare centres and provides only one parameter particularly sensitive to TBA changes thus representing a suitable tool for an easy and fast applicability into both research and clinical practice. MRI systems with 1.5 T magnetic field power provide high resolution images with pixel size of about 400 μm. As already discussed elsewhere [14], while Tb. Th is smaller than such a resolution (100–300 μm), bone marrow spaces are larger (averagely 800–2000 μm) thus making still reasonable TBA characterisation by MRI [13, 47]. In addition, image processing and image analysis techniques allow overcoming the limits of image quality and resolution. The computational approach adopted in our method to quantify TBA deterioration by TBLβ, based on fractal lacunarity texture analysis in greyscale images, overcomes the limits of image binarization process and provides one parametric result representative of an holistic characterisation of TBA, comprehensive of BV/TV, Tb. Sp, Tb. N, Tb.Th.

Baseline results from LOTO study confirm the goodness of TBLβ as an index of osteoporotic fracture risk more suitable than BMD in separate patients with and without bone fragility fractures. Introducing TBA characterisation by TBLβ into clinical practice to complement DXA-BMD based diagnosis would contribute to identify up to 86% women at high risk of bone fragility fracture. Preliminary results from 1-year follow up seem to be even more promising. The projection of AR, that is the risk due to the single risk factor considered, suggests that by monitoring TBA degeneration as a risk factor for bone fragility fracture would reduce the incidence of osteoporotic fractures of 25% vs. only 6% for BMD.

It is worth noting that differences in results between whole sample and untreated group forewarn the potential of TBLβ as an index useful in the assessment of therapy efficacy. Preliminary results previously presented [56] further support the potential role TBLβ can play in monitoring the efficacy of osteoporosis drug treatment in preventing or reducing bone fragility fracture risk. This aspect needs a deeper analysis of baseline data as they are from an observational study with heterogeneity in both therapy type and time. We expect more consistent results about this aspect from LOTO study prospective phases as information on both type and time of osteoporosis medications prescribed to baseline untreated patients would be well documented. Nevertheless, dedicated pharmacologic studies are also necessary.

This study represents a first step toward clinical validation of the proposed method. Multicentric studies are desirable and phantom based setup of different MRI instrumentations from several health centres will need. It would allow establishing a common protocol of image acquisition to guarantee comparison of results by avoiding discrepancies among different manufacturers devices.

In this context, the proposed method, based on fractal lacunarity texture analysis of MRI-TBA, is easy and fast to apply thus making simple to face this kind of studies.

Improvements of the method are in progress to overcome the limit of rectangular ROI, used in this study, by adopting a circle-like shape. A circular ROI to fit the whole trabecular bone area within the inner perimeter of vertebral body would allow for a more accurate quantification of TBA deterioration.

## Conclusion

TBLβ method, based on fractal lacunarity of TBA in vertebral MRI, represents a useful noninvasive/nonionizing tool to assess TBA deterioration and predict bone fragility fracture. TBLβ has also potential as an index to monitor osteoporosis therapy response. TBLβ method uses 1.5 T-MRI, widely available in clinical setting, and provides only one parameter, TBLβ, to characterise TBA, thus representing an easy and fast tool promptly applicable to both clinical and research studies on osteoporosis.

## Data Availability

The datasets generated and analyzed during the current study are available from the corresponding author upon reasonable request.

## Abbreviations

ACC: Accuracy
AR: Attributable risk
AUC: Area under the curve
BMD: Bone mineral density
BV/TV: Bone volume fraction
CT: Computer tomography
DXA: Dual-energy x-ray absorptiometry
FRAX: Fracture risk assessment tool
GBA: Gliding box algorithm
GTA: Geodesic topological analysis
HR-pQCT: High resolution-peripheral quantitative CT
LOTO: Lacunarity Of Trabecular bone in Osteoporosis
MRI: Magnetic resonance imaging
MRX: Morphometry on conventional x-ray images
MXA: Morphometry on densitometric images
NPV: Negative predictive value
OR: Odds ratio
PPV: Positive predictive value
ROC: Receiver Operating Characteristics
RR: Relative risk
SN: Sensitivity
SP: Specificity
T+: treated subjects
T-: Untreated subjects
TBA: Trabecular bone microarchitecture
TBLβ: Trabecular bone lacunarity parameter β
Tb.N: Trabecular bone number
Tb.Sp: Trabecular bone spacing
Tb.Th: Trabecular bone thickness
VF: Vertebral fracture
VF+: with prevalent vertebral fractures.
VF-: Without vertebral fractures
WHO: World Health Organization
